# Thermal Stress and Coral Cover as Drivers of Coral Disease Outbreaks 

**DOI:** 10.1371/journal.pbio.0050124

**Published:** 2007-05-08

**Authors:** John F Bruno, Elizabeth R Selig, Kenneth S Casey, Cathie A Page, Bette L Willis, C. Drew Harvell, Hugh Sweatman, Amy M Melendy

**Affiliations:** 1 Department of Marine Sciences, The University of North Carolina at Chapel Hill, Chapel Hill, North Carolina, United States of America; 2 Curriculum in Ecology and Department of Marine Sciences, The University of North Carolina at Chapel Hill, Chapel Hill, North Carolina, United States of America; 3 National Oceanographic Data Center, National Oceanic and Atmospheric Administration, Silver Spring, Maryland, United States of America; 4 Australian Research Council Centre of Excellence in Coral Reef Studies, School of Marine and Tropical Biology, James Cook University, Townsville, Queensland, Australia; 5 Section of Ecology and Evolutionary Biology, Cornell University, Ithaca, New York, United States of America; 6 Australian Institute of Marine Science, Townsville, Queensland, Australia; 7 Department of Epidemiology, The University of North Carolina at Chapel Hill, Chapel Hill, North Carolina, United States of America; University of York, United Kingdom

## Abstract

Very little is known about how environmental changes such as increasing temperature affect disease dynamics in the ocean, especially at large spatial scales. We asked whether the frequency of warm temperature anomalies is positively related to the frequency of coral disease across 1,500 km of Australia's Great Barrier Reef. We used a new high-resolution satellite dataset of ocean temperature and 6 y of coral disease and coral cover data from annual surveys of 48 reefs to answer this question. We found a highly significant relationship between the frequencies of warm temperature anomalies and of white syndrome, an emergent disease, or potentially, a group of diseases, of Pacific reef-building corals. The effect of temperature was highly dependent on coral cover because white syndrome outbreaks followed warm years, but only on high (>50%) cover reefs, suggesting an important role of host density as a threshold for outbreaks. Our results indicate that the frequency of temperature anomalies, which is predicted to increase in most tropical oceans, can increase the susceptibility of corals to disease, leading to outbreaks where corals are abundant.

## Author Summary

Coral reefs have been decimated over the last several decades. The global decline of reef-building corals is of particular concern. Infectious diseases are thought to be key to this mass coral mortality, and many reef ecologists suspect that anomalously high ocean temperatures contribute to the increased incidence and severity of disease outbreaks. This hypothesis is supported by local observations—for example, that some coral diseases become more prevalent in the summertime—but it has never been tested at large spatial scales or over relatively long periods. We tested the temperature–disease hypothesis by combining 6 years of survey data from reefs across 1,500 kilometers of Australia's Great Barrier Reef with a new ocean temperature database derived from satellite measurements. Our results indicate that major outbreaks of the coral disease white syndrome only occurred on reefs with high coral cover after especially warm years. The disease was usually absent on cooler, low-cover reefs. Our results suggest that climate change could be increasing the severity of disease in the ocean, leading to a decline in the health of marine ecosystems and the loss of the resources and services humans derive from them.

## Abstract 3

High-resolution satellite data of ocean temperature across the Great Barrier Reef reveal that warm temperature anomalies can drive outbreaks of coral disease under conditions of high coral cover.

## Introduction

Climatic and oceanographic conditions can modify a wide variety of ecological processes. For example, ocean temperature can control species ranges, the strength of species interactions, the dispersal and survival of marine larvae, and the rates of metabolism and speciation [1–6]. Additionally, anomalously high temperature and other environmental stresses can influence the severity and dynamics of infectious diseases by increasing host susceptibility and pathogen virulence [7,8]. For example, the severity of human epidemics including cholera [9–11] and tick-borne encephalitis [12] are both related to temperature and, possibly, to recent climate change [13]. Temperature and climate change have also been implicated in plant and animal disease outbreaks in both terrestrial and aquatic habitats [7,14–17], and could influence coral disease severity [18–20], potentially accelerating the global loss of coral reefs.

Corals are the foundation species of tropical coral reef ecosystems. They directly facilitate thousands of associated species by generating the physically complex reef structure [21,22]. Reductions in coral abundance can cause rapid loss of reef biodiversity [23]. The hypothesized link between anomalously high temperatures and coral disease outbreaks is supported by small-scale field studies indicating that prevalence and the rate of within-colony spread of several coral diseases are higher during the summer [24–30]. Such seasonal changes in disease severity could be driven in part by higher summertime temperature, but could also be caused by a variety of other abiotic factors that vary seasonally within sites. Additionally, such investigations do not directly address the role of temperature anomalies in driving the conspicuous variability of coral disease severity among years and locations [30–32] that has long intrigued coral reef ecologists. Missing are large-scale, longitudinal investigations that combine long-term monitoring of multiple populations with accurate, fine-grained measurements of local temperature anomalies. Longitudinal studies (i.e., the repeated sampling of individuals or populations) help control for potential confounding factors and inherent temporal variability [33]. Such powerful epidemiological approaches are rarely applied to marine epidemics (but see [34,35]), which has limited our understanding of potential links between temperature and disease outbreaks in the ocean, especially at large spatial scales.

Here we describe a regional-scale test of the hypothesis that ocean temperature can influence disease frequency. We analyzed the relationship between the frequency of white syndrome in scleractinian corals and of warm temperature anomalies across the Great Barrier Reef (GBR). Forty-eight reefs were monitored for 6 y (1998–2004), and reef-specific weekly sea surface temperature anomalies (WSSTAs; the frequency of deviations ≥ 1 °C) were derived from a satellite sea surface temperature (SST) database. White syndrome is an emerging disease of Pacific reef-building corals, reported in 17 species from families including Acroporidae, Pocilloporidae, and Faviidae, which comprise the majority of dominant species on the GBR [30]. Severe white syndrome outbreaks can affect coral composition and cover [30]. Little is known about the etiology of white syndrome, although it is presumably infectious and the characteristics are similar to Caribbean white diseases such as white band and white plague [36]. Like the Caribbean white diseases, white syndrome could comprise a group of distinct diseases with similar signs [30]. White syndrome can cause either partial or whole colony mortality and is characterized by a white band of tissue or recently exposed skeleton that moves across the colony as the disease progresses [30,37].

## Results

**Figure 1:**
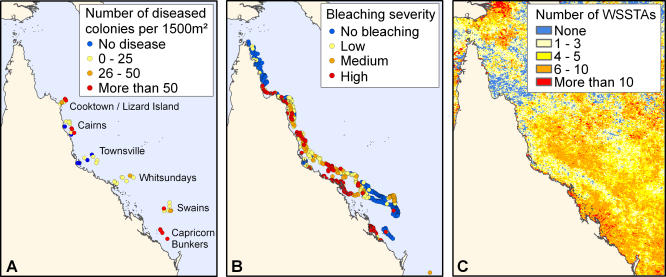
Study Sites and Disease Conditions during the Peak of White Syndrome Frequency in 2002 (A) Frequency of white syndrome cases from March 2002 to March 2003; (B) bleaching intensity for scleractinian coral in March 2002 (modified from Berkelmans et al., 2004 [73]); and (C) WSSTAs in 2002.

White syndrome has been present on the GBR since at least the beginning of systematic disease monitoring in 1998, but its frequency increased 20-fold in 2002 [30]. This rise came after a year in which the region experienced its second warmest summer in the 20-y satellite record, with 58% of reefs having weekly anomalies of 1 °C or higher. However, even during the peak of the outbreak, there was considerable variation in disease frequency among reefs (0 to 343 cases per 1,500 m^2^) (Figure 1B). WSSTA also varied substantially among reefs, especially during the warm summers of 1998/1999 and 2001/2002 when some reefs were anomalously warm for 30 wk of the year, but the weekly temperatures on many others never deviated from the long-term local averages (i.e., WSSTA = 0).

Reefs with relatively high coral cover and WSSTA had the greatest white syndrome frequency (Table 1). From the negative binomial regression model, the parameter estimates for the three covariates (WSSTA, coral cover, and the interaction between the two) were positive (i.e., they predicted an increase in frequency) and highly significant (all *p* < 0.000; Table 2). The interaction term (WSSTA × coral cover) explained a statistically significant amount of the increase in frequency of disease among all the covariates in the model (χ^2^ = 17.49, *df* = 1, *p* < 0.0000). The deviance statistic for the negative binomial model was 1.0201, suggesting a very good fit to the data. Disease frequency predicted for nine WSSTA–coral cover combinations (based on the tenth, 50th, and 90th observed quintiles of these covariates) is presented in Table 3. The observed and predicted values indicate that disease frequency only increases substantially with the combination of extreme levels of both covariates. The model is a fairly conservative predictor of this relationship because the observed number of cases with high WSSTA and high coral cover (Table 1) was actually higher than predicted by the model.

**Table 1:**
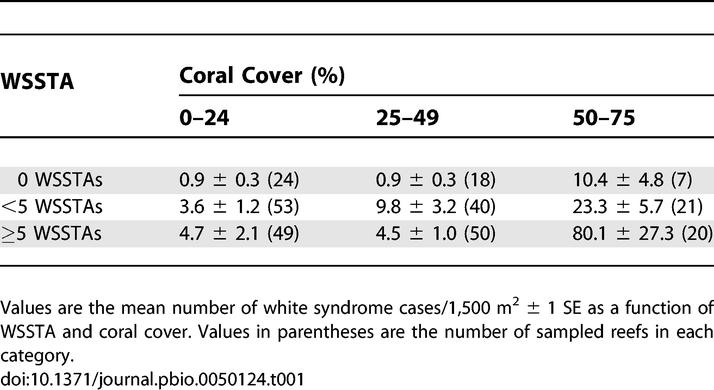
Observed Number of White Syndrome Cases

**Table 2:**
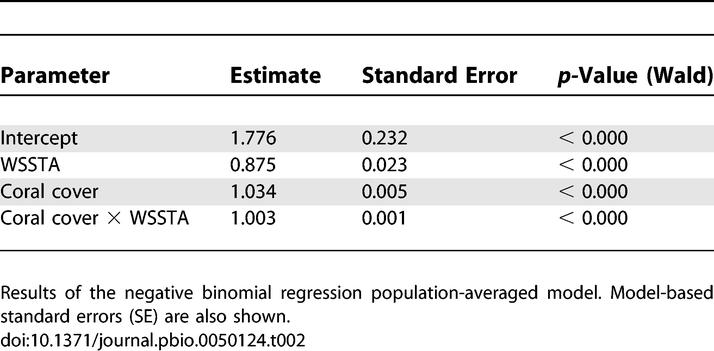
Coefficient Estimates

**Table 3:**
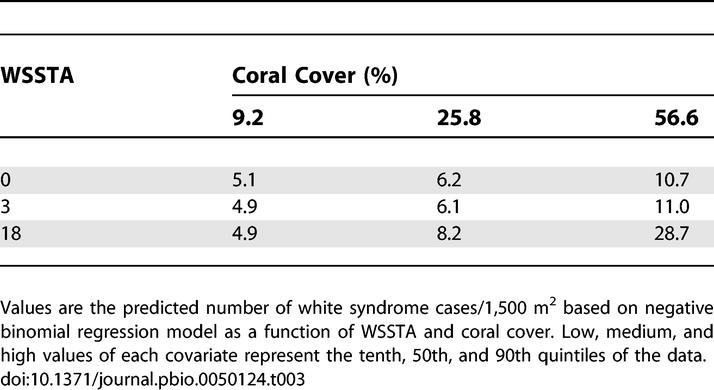
Predicted Number of White Syndrome Cases

## Discussion

### Influence of Warm Temperature Anomalies

The frequency of warm temperature anomalies was positively related to white syndrome frequency across the GBR. The disease surveys documented considerable variation in white syndrome frequency (0 to 343 cases per 1,500 m^2^) among years and reefs. Our results suggest that this variance was caused in part by the number of warm temperature anomalies during the year preceding the disease surveys. A positive effect of high temperature on the severity of coral disease outbreaks might be caused by physiological stress impairing host immunity [8,38,39]. WSSTA, our metric of thermal stress, is based on the frequency of warm anomalies of 1 °C or higher because short-term temperature increases of this degree can cause measurable physiological stress in a coral host [40–46]. WSSTA summarized temperature anomalies throughout the year, including winter anomalies that might also affect the susceptibility of corals to disease [46]. Increased densities of symbiotic dinoflagellate algae (zooxanthellae) at the beginning of winter and the subsequent accumulation of coral-tissue biomass throughout cooler months are thought to influence coral responses to future stresses [47]. These processes are compromised by longer warm periods during the summer or warmer than usual winter temperatures [45]. In fact, winter warming could have the dual effect of predisposing hosts to disease and facilitating more rapid pathogen growth [7]. Summertime anomalies could also increase pathogen virulence by initiating virulence factors [48] or increasing the growth of pathogens [39] for which the normal summertime temperature is below the thermal optima.

### Influence of Coral Cover

**Figure 2:**
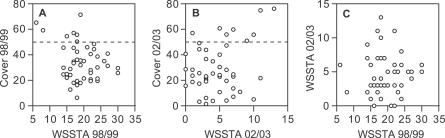
Relationship between Coral Cover and Thermal Stress (A and B) Relationships between thermal stress (number of WSSTAs) and total scleractinian coral cover during the two warmest summers of the study. Dashed line represents the empirical cover threshold of 50% that is generally required for high white syndrome frequency. (C) Relationship of thermal stress between the summers of 1998/1999 and 2002/2003 at 48 reefs on the GBR.

Our results also indicate that thermal stress is necessary, but not sufficient, for white syndrome outbreaks to occur. Coral cover must also be high; generally 50% or higher (Table 1). White syndrome was uncommon during the 12 mo after the summer of 1998/1999 when WSSTAs were more frequent and occurred at more sites than during 2002/2003. But in 1998/1999, total coral cover was less than 50% at the 20 reefs with the highest WSSTA (Figure 2A), and there was a weak negative relationship between WSSTA and cover (*p* = 0.09, linear regression analysis; Figure 2A). In contrast, in 2002/2003, there was a positive association between WSSTA and coral cover (*p* = 0.05, Figure 2B). This was possible because there was no reef-specific correlation of WSSTA between 1998/1999 and 2002/2003 (*p* = 0.90, Figure 2C).

Total coral cover is a reasonable estimation of host abundance in this system because the susceptible species are the competitively dominant space holders [30,49]. A positive relationship between host density and disease prevalence has been clearly demonstrated in many host-pathogen systems [35,50–52], and is considered a hallmark of the infectious process [53]. High host density can have several effects on disease dynamics. For example, it is most often associated with greater rates of horizontal transmission [54–56], leading to localized increases in prevalence. High coral cover reduces the distance between neighboring coral colonies [57] and thus between infected and healthy hosts, increasing the potential for horizontal disease transmission between corals in close proximity. In addition, host density can be positively related to the density of disease vectors [58,59], although no specific vector(s) have been identified for white syndrome.

Independent of host density, total coral cover itself, including the abundance of nonsusceptible individuals and species, might also be causally linked with increased white syndrome frequency. A wide variety of biological properties of coral reefs are related to coral cover. For example, the abundance and composition of fishes and invertebrates that could act as disease vectors are tightly linked with total coral cover and reef heterogeneity [21,23]. Competitive interactions among corals increase nearly exponentially with total coral cover and, on the GBR, are relatively rare when cover is below 50% [57]. Corals compete directly by damaging the tissue of neighboring colonies with tentacles and digestive filaments [60]. These encounters usually cause lesions and local tissue necrosis [60] that could facilitate pathogen transmission and colony infection. Additionally, uninfected hosts likely experience physiological stress and a reduction in fitness on high-cover reefs from such direct competitive encounters [61] as well as from indirect competition such as shading [60], which could also reduce disease resistance.

Regardless of the relative importance of these and other potential mechanisms for increased host susceptibility or disease transmission where coral cover is high, there is a cover threshold of approximately 50% (Table 1) for white syndrome outbreaks and, frequently, even for the occurrence of this disease on a reef. No white syndrome cases were recorded on 45% of the reefs with cover less than 50% (*n* = 235). In contrast, 88% of reefs with cover greater than 50% had at least one infected colony (*n* = 47). Such thresholds for pathogen colonization or persistence based on host density or other factors are theoretically predicted and typical of the dynamics of many wildlife diseases [8,35].

The technique used to measure the intensity of community-wide white syndrome outbreaks (i.e., counting the number of infected colonies) could lead to a spurious relationship between coral cover and disease frequency, since more colonies could be sampled at higher-cover reefs. This potential artifact was accounted for by including coral cover as a covariate in the statistical model. Additionally, our results indicate that this potential sampling effect did not occur, or at least was undetectable. For example, disease frequency is very low and essentially constant across reefs with coral cover ranging from 0% to 50% (Table 1). Furthermore, the significant WSSTA × coral cover interaction term indicates that the coral cover effect was nonadditive. Finally, on the GBR and other Indo-Pacific reefs, coral cover and colony density generally are not positively related [62]. During the early stages of reef recovery after a major disturbance when nearly all colonies are small and coral cover is very low [62], colony density and cover can be positively related [57,63]. However, when coral cover is high, reefs are usually dominated by large colonies [62], and density and cover are typically negatively related [57]. On the GBR, this frequently observed parabolic relationship between coral cover and colony density is caused by the domination of high-cover reefs by large tabular colonies that exclude smaller non-tabulate species [49]. This was the case in our study on the highest cover reefs in the Cooktown/Lizard Island and Capricorn Bunkers sectors (Figure 1A) where most of the white syndrome outbreaks occurred (C. Page, personal communication). Therefore, our sampling design could in fact *underestimate* disease severity on very high-cover reefs, diminishing the measured importance of coral cover.

### Ocean Temperature and the Impacts of Disease

Diseases can cause dramatic changes in host populations and can have lasting effects on the structure and functioning of marine ecosystems by reducing the abundance of keystone consumers and habitat-forming foundation species [19,53,64,65]. For example, a pandemic wasting disease of eelgrass populations in the 1930s caused widespread losses along the Atlantic coasts of Canada, the United States, and Europe [66]. In some affected areas, the disease was estimated to have reduced stands to less than 1% of their normal abundance [67]. Oyster diseases in the Chesapeake Bay, where the pathogen Perkinsus marinus has caused annual mortality ranging from 24% to 57%, contributed to the commercial collapse of the regional oyster industry and to the regional loss of oyster reef habitat [68]. Similarly, an unidentified disease decimated populations of the keystone herbivore Diadema antillarum in the 1980s throughout the Caribbean [69,70]. During the same time period, white band disease dramatically reduced the abundance of the two most abundant Caribbean corals, Acropora palmata and *A. cervicornis,* causing changes in reef structure unprecedented in the last 3,000 y [71,72].

The impacts of marine epidemics could increase if warm temperature anomalies become more frequent or extreme [13,18,19] as predicted by several climate change models [41]. Additionally, temperature could have locally additive or even synergistic impacts if the prevalence of disease or multiple diseases and non-infectious bleaching is increased by warm temperature anomalies [29,38]. For example, bleached corals could be more susceptible to infection [38]. The peak of the white syndrome outbreak occurred after the very warm austral summer of 2001/2002, concomitant with the most severe bleaching episode—in terms of number of reefs affected and intensity of bleaching—ever recorded on the GBR [73]. On some reefs, bleaching and outbreaks of atramentous necrosis, another GBR coral disease, occurred nearly simultaneously [29]. But surprisingly, across the GBR, there was little spatial concordance between bleaching and white syndrome severity in 2001/2002. The most intensive bleaching during 2001/2002 was concentrated in the central latitudes [73] where white syndrome frequency was generally very low (Figure 1). In contrast, there was little or no bleaching on reefs in the southern GBR, including the Capricorn Bunkers sector, where white syndrome outbreaks were most severe (Figure 1). The causes of this negative correlation are unclear, but could include variable host susceptibility, local species composition, thermal history, and prior disturbances. Regardless of potential causes, the segregation of these two impacts of anomalously high temperatures might limit local coral loss, but could lead to additive net declines in coral cover across the region.

**Figure 3:**
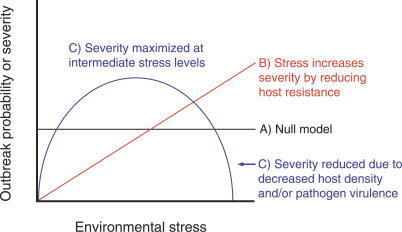
Predicted Effects of Environmental Stress on Disease Severity Conceptual model of potential effects of environmental stress (magnitude or frequency) on the probability or severity (e.g., prevalence or impacts on host populations) of disease outbreaks. The model includes three possible scenarios: (A) the null model of no effect, (B) a positive, linear effect of stress such as when host density is unrelated to incidence and when the pathogen is not negatively affected by the stress, and (C) a parabolic stress effect.

Alternatively, rising ocean temperature or an increase in summertime anomalies could inhibit marine epidemics. Environmental stress is often assumed to increase disease severity, but stresses that directly reduce host density can have the opposite effect [8] (Figure 3). The role of coral cover in mediating the influence of temperature on disease frequency suggests that temperature could have an important inhibitory effect on white syndrome via bleaching-induced coral mortality. High temperatures only 1–2 °C above the normal summer maximum can cause bleaching and mass coral mortality [41,42], leading to a reduction in host density and total coral cover. Therefore, anomalously high water temperature could, in contrast to our results, reduce the prevalence of coral diseases with host density or coral cover thresholds. However, host density is not always related to the spread of disease, such as when the disease is not infectious, if local secondary transmission is rare, and when pathogens originate outside the local host population or in other host species [8]. In such cases, the relationship between stress and disease severity is generally predicted to be positive [8] (Figure 3).

Environmental stress can also reduce the intensity or probability of outbreaks by negatively affecting pathogen fitness or virulence [8]. It is possible that coral pathogens are negatively affected by anomalously high water temperature. In fact, laboratory studies have found that beyond thermal optima, coral pathogens can have reduced photosynthetic [74] and growth rates [39]. Direct, negative effects of environmental stresses on either hosts or pathogens could cause a parabolic relationship between the magnitude or frequency of environmental stresses and disease incidence, with outbreaks occurring mainly at intermediate stress levels (Figure 3). Thus, future increases in thermal anomalies or other forms of environmental stress could decrease the probability and severity of marine epidemics.

Paradoxically, management activities that increase host abundance could facilitate epidemics. Indeed, most of the major coral reef epidemics over the last 20 y have been of high-density hosts. Caribbean examples include acroporid white band disease outbreaks [71], the D. antillarum epidemic [69,70], and sea fan aspergillosis [75]. Once the density of these hosts was sufficiently diminished, prevalence often decreased [34], and host populations began to recover [76,77].

### Conclusions

Warm temperature anomalies and coral cover are clearly important drivers of white syndrome on the GBR. No previous study has demonstrated a link between ocean temperature and coral disease dynamics, especially at regional spatial scales. Our results are supported by basic epidemiological principles, and could apply to other coral disease systems and to disease ecology in general. However, coral disease dynamics are likely to be affected by a variety of biotic and abiotic factors, the relative importance of which will vary among regions, scales, and species [32]. In some locations, coral disease outbreaks are apparently decoupled from temperature, and several other factors are also known or suspected to influence the dynamics of coral and other marine diseases [19]. For example, the severity of at least three coral diseases is linked with nutrient concentrations [25,32,75], whereas the frequency of others, like white syndrome, is greatest on remote reefs in highly oligotrophic waters [30].

Coral reefs around the world have been dramatically transformed over the last several decades as coral cover decreased and reefs became dominated by macroalgae [71,78–80]. These changes affect entire coral reef ecosystems, resulting in declines in biodiversity, fisheries yield, and other ecosystem services [81]. Our results indicate warm temperature anomalies can drive outbreaks of coral disease under conditions of high coral cover. The general increase in coral disease prevalence and the emergence of several new coral diseases over the last two decades [20,82,83] could also have been caused in part by thermal anomalies. Deciphering these and other effects of increasing temperature on disease dynamics in the ocean presents an urgent challenge to marine scientists.

## Materials and Methods

### Disease and coral cover surveys.

Surveys of white syndrome frequency and total coral cover (i.e., the percentage of the bottom covered by living scleractinian corals) were performed by the Australian Institute of Marine Science Long-term Monitoring Program. The 48 surveyed reefs are grouped within six latitudinal sectors that span nearly 1,500 km of the GBR from 14° S to 24° S (Figure 1A). Surveys were performed annually between 1998 and 2004 using SCUBA along a depth contour of 6 to 9 m on the northeast flank of each reef. The frequency of white syndrome cases on each reef (number/1,500 m^2^) was measured by counting the number of infected colonies within 15 permanent 50-m × 2-m belt transects [30,84]. The percentage of the substrate covered with living, hard (scleractinian) coral tissue was quantified on 15 permanent 50-m transects, within a 25-cm-wide belt along the transect using a video camera [85]. A point sampling technique was then used to estimate live coral cover from the videos in the laboratory [85].

### Satellite temperature database and thermal stress metric.

We derived weekly sea surface temperature values for each reef from a newly developed 4-km Advanced Very High Resolution Radiometer Pathfinder temperature anomaly dataset (Version 5.0) developed by the National Oceanic and Atmospheric Association and the University of Miami's Rosenstiel School of Marine and Atmospheric Science. This dataset covers the longest time period (1985–2004) at the highest resolution of any consistently processed, global satellite temperature dataset. We used nighttime, weekly-averaged values with a quality level of four or better [86]. Some plausible values were given low-quality levels by the Pathfinder algorithm, which eliminates any observation with an SST more than 2 °C different than a relatively coarse resolution SST field based on the Version 2 Reynolds Optimum Interpolation Sea Surface Temperature (OISST) value, a long-term, in situ–based dataset [86,87]. Therefore, we included observations if the SST was greater than the OISST, but less than the OISST + 5 °C. Gaps in the record caused by persistent cloudiness were filled using simple temporal interpolation to provide a complete weekly time series at each reef spanning 1985–2004.

We generated a 19-y, weekly SST climatology (i.e., a long-term record) for the 4 × 4-km grid cell that encompassed each reef. A 5-wk running mean was then used to smooth each gap-free climatology to minimize any unusual fluctuations caused by periods of limited data availability. Although thermal stress metrics have been created to predict bleaching events from satellite SST data [73,88,89], little is known about the thresholds relevant to coral disease. In general, increases of 0.5–1.5 °C for several weeks can induce coral bleaching [42]. We assumed that temperatures that may lead to bleaching and physiological stress in corals [42,45] could also potentially increase susceptibility to disease [7].

In a pilot study, we created 16 different metrics of thermal stress. After initial screening, Akaike Information Criteria (AIC) identified WSSTA as the metric that best explained the relationship between temperature and disease (three of the other metrics and the selection procedure are described in [37]). WSSTA quantified the frequency of high-temperature anomalies experienced by coral hosts and by the potential white syndrome pathogen(s), during the 52 wk prior to the annual disease surveys. WSSTAs represent the number of annual deviations of 1 °C or higher from a mean climatology calculated from records between 1985 and 2004 for that calendar week at that reef. Thus, the metric is both week specific and location specific, and considers deviations from local climatological averages, i.e., typical SST throughout the year, including wintertime high-temperature anomalies that could also influence coral fitness and susceptibility to infection [46]. Because recent field and laboratory studies indicate that corals on the GBR are significantly adapted to local thermal conditions [43,90], we based WSSTA on the local SST climatology created independently for each of the 48 reefs. Furthermore, our long-term, fine-grained measurements of SST and SST anomalies match the scale of the biological surveys, eliminating the usual mismatch between climate and health data that has plagued similar studies of human and wildlife disease dynamics [91].

### Statistical analysis.

We used negative binomial regression to model the relationship between thermal stress and coral cover and the frequency of white syndrome cases (i.e., the number/1,500 m^2^). Negative binomial regression was ideal for this analysis because the dependent variable was continuous and overdispersed (i.e., the variance exceeds the mean). The covariates in the model included WSSTA, coral cover, and the interaction term, which represents the multiplicative relationship between coral cover and temperature. Because there is a biologically plausible mechanism by which an interaction between coral cover and temperature could affect the overall outcome (i.e., the influence of thermal stress could be coral cover dependent), it was important to include this interaction as a covariate. A host density threshold is a common signature of infectious disease outbreaks of humans and other marine taxa such as viral diseases of seals [50,52,53]. Total coral cover or the abundance of susceptible species could both influence disease frequency and the effect of temperature on frequency. Coral cover was also included in the model to account for the potential positive relationship between cover and disease frequency based solely on the fact that the number of surveyed colonies may have increased with coral cover.

Because the individual sampling units (reefs) were nested within larger groupings (sectors), this factor was included as a stratification variable to control for the main effect of variance within and between sectors. We used the general estimating equations (GEE) (i.e., population averaged) to estimate parameters of the negative binomial model, which accounted for the repeated measurement of the individual sampling unit (reefs, each sampled once a year for six consecutive years). An autocorrelative structure was initially included; however, the parameter was sufficiently close to zero (0.01 ± 0.05 standard error [SE]) to consider the autocorrelative effects negligible, and thus was not included in the final model. We also calculated a deviance statistic (i.e., deviance/degrees of freedom) to assess the goodness of fit of the model. If the model and the designated distribution are correct, this value should be approximately 1.0.

Many longitudinal datasets with continuous dependent variables are modeled using Poisson regression [33]. However, the variance structure of the related regression model, the negative binomial, includes a random dispersion term and is thus more flexible and appropriate in assessing the relationship between the covariates and an overdispersed dependent variable [92,93]. We did run a Poisson regression model, and the deviance statistic was 24.4203, indicating a poor fit to the data. Zero inflation, that is, the possibility of the existence of a population of hosts for which the outcome cannot happen (e.g., reefs with no susceptible individuals), was also of potential concern. To address this issue, we fit zero-inflated negative binomial and Poisson regression models. There was no difference in parameter estimates from the standard models; thus, the simplest negative binomial model was used in the final analysis. All regression analyses were conducted using Intercooled Stata 9.1 (Stata Corporation, http://www.stata.com).

## Abbreviations

GBR: Great Barrier Reef
OISST: Optimum Interpolation Sea Surface Temperature
SST: sea surface temperature
WSSTA: weekly sea surface temperature anomaly
